# Early host immune responses in a human organoid-derived gallbladder monolayer to *Salmonella* Typhi strains from patients with acute and chronic infections: a comparative analysis

**DOI:** 10.3389/fimmu.2024.1334762

**Published:** 2024-03-12

**Authors:** Rosângela Salerno-Goncalves, Haiyan Chen, Andrea C. Bafford, Mariana Izquierdo, Juan Carlos Hormazábal, Rosanna Lagos, Hervé Tettelin, Adonis D’Mello, Jayaum S. Booth, Alessio Fasano, Myron M. Levine, Marcelo B. Sztein

**Affiliations:** ^1^ Center for Vaccine Development and Global Health and Department of Pediatrics, University of Maryland School of Medicine, Baltimore, MD, United States; ^2^ Division of General and Oncologic Surgery, University of Maryland School of Medicine, Baltimore, MD, United States; ^3^ Seccion Bacteriologia, Subdepartamento de Enfermedades Infecciosas, Departamento de Laboratorio Biomédico, Instituto de Salud Pública de Chile (ISP), Santiago, Chile; ^4^ Department of Microbiology and Immunology and Institute for Genome Sciences (IGS), University of Maryland School of Medicine, Baltimore, MD, United States; ^5^ Mucosal Immunology and Biology Research Center, Massachusetts General Hospital for Children, Boston, MA, United States; ^6^ Program in Oncology, University of Maryland Marlene and Stewart Greenebaum Comprehensive Cancer Center, Baltimore, MD, United States

**Keywords:** bacteria, Salmonella, human, chronic infection, gallbladder

## Abstract

*Salmonella enterica* serovar Typhi (*S*. Typhi), a human-restricted pathogen, invades the host through the gut to cause typhoid fever. Recent calculations of the typhoid fever burden estimated that more than 10 million new typhoid fever cases occur in low and middle-income countries, resulting in 65,400-187,700 deaths yearly. Interestingly, if not antibiotic-treated, upon the resolution of acute disease, 1%-5% of patients become asymptomatic chronic carriers. Chronically infected hosts are not only critical reservoirs of infection that transmit the disease to naive individuals but are also predisposed to developing gallbladder carcinoma. Nevertheless, the molecular mechanisms involved in the early interactions between gallbladder epithelial cells and *S*. Typhi remain largely unknown. Based on our previous studies showing that closely related *S*. Typhi strains elicit distinct innate immune responses, we hypothesized that host molecular pathways activated by *S*. Typhi strains derived from acutely and chronically infected patients would differ. To test this hypothesis, we used a novel human organoid-derived polarized gallbladder monolayer model, and *S*. Typhi strains derived from acutely and chronically infected patients. We found that *S*. Typhi strains derived from acutely and chronically infected patients differentially regulate host mitogen-activated protein kinase (MAPK) and S6 transcription factors. These variations might be attributed to differential cytokine signaling, predominantly via TNF-α and IL-6 production and appear to be influenced by the duration the isolate was subjected to selective pressures in the gallbladder. These findings represent a significant leap in understanding the complexities behind chronic *S*. Typhi infections in the gallbladder and may uncover potential intervention targets.

## Introduction

Typhoid fever caused by *Salmonella enterica* serovar Typhi (*S.* Typhi), is a significant public health problem. In industrialized countries, typhoid fever is rare. Most infections among high income countries occur among individuals traveling to endemic areas (including military personnel) and laboratory workers. Yet, more than 10 million new cases of typhoid fever occur in low and middle-income countries, with about 65,400-187,700 deaths per year (1–5). The disease spreads through the fecal-oral route via contaminated food and water (6), invades the distal ileum, and disseminates systemically to cause typhoid fever (7). Depending on the choice of antibiotic used to treat acute typhoid and the susceptibility of the infecting strain, 1-5% of patients can become long-term chronic gallbladder carriers of *S*. Typhi (8, 9). These individuals intermittently shed large numbers of *S*. Typhi in their stools and are at an increased risk of developing gallbladder carcinoma (GBC) (10, 11). Epidemiological associations between GBC and typhoid carriage have been reported in some typhoid-endemic areas (12–16). GBC is highly lethal, with a 5-year survival of approximately 12% (10).

In addition, while antibiotics had drastically decreased the morbidity and mortality rates of typhoid fever in both adults and children, the emergence of antibiotic resistance in *S*. Typhi has reignited the need for new therapeutics, fueling renewed enthusiasm in the study of chronic carriers. In this regard, a recent study using murine organoid-derived gallbladder monolayer has shown that *S*. Typhimurium-mediated activation of Akt and mitogen-activated protein kinase (MAPK) pathways drives the malignant transformation of susceptible cells (11). Moreover, Sepe and colleagues have demonstrated that *S*. Paratyphi A typhoid toxin can cause genome instability in primary epithelial cells derived from a human gallbladder organoid (17). Nevertheless, the early molecular mechanisms involved in the interactions between human gallbladder cells and *S.* Typhi are poorly understood. In addition, there are no reports on the possible differential activation of AKT and MAPK pathways by *S*. Typhi strains derived from acutely and chronically infected patients.

We hypothesize that host early molecular pathways activated by *S*. Typhi strains derived from acutely and chronically infected patients will differ. This hypothesis is based on our previous studies demonstrating that very closely related *S*. Typhi strains can elicit different innate cell responses in the human intestinal mucosa (18, 19). To test his hypothesis, we used a human organoid-derived gallbladder monolayer (HODGM) model developed by our group, and 22 *S*. Typhi strains derived from patients with acute typhoid fever (n=11) versus strains from persons with chronic (n=11) *S*. Typhi gallbladder infection. *S.* Typhi is a human-restricted pathogen, and no animal models faithfully recapitulate *S*. Typhi infection (20, 21). Herein, we found that *S*. Typhi strains derived from acutely and chronically infected patients differentially regulate MAPK and S6 transcription factors. This differential regulation impacts, at least in part, the cytokine signaling pathway involved in the production of TNF-α and IL-6. Our research also underscores a potential link between TNF-α levels and the duration a clinical isolate experiences selective pressure within the human gallbladder.

## Materials and methods

### Ethics statement

Anonymized leftover gallbladder tissues were collected from patients undergoing laparoscopic cholecystectomy for biliary colic or chronic cholecystitis. A protocol describing the collection and use of these samples has been submitted to the University of Maryland Institutional Review Board (IRB), and a study exemption has been approved (#HP-00077485). All authors had access to the study data and reviewed and approved the final manuscript.

### Bacterial strain, media, and growth conditions

This manuscript utilizes an archived collection of 22 unique *S*. Typhi strains isolated from both acutely (n=11) and chronically (n=11) infected patients (**Supplementary Table 1**), which were cryopreserved and stored in our Cryo-Bank Cell Repositories. The strains obtained from acutely infected patients include Ty2 (22), the parent strain of the oral typhoid vaccine Ty21a (23), along with 10 strains obtained from Santiago, Chile, during two distinct periods: the 1980s (n=5) (24, 25) and between 2017 and 2019 (n=5) (26). The strains isolated from chronically infected patients consist of Quailes (27), as well as 10 strains also obtained from public hospitals in Santiago during the 1980s (n=6) and between 2017 and 2019 (n=4) (26). Chile has high GBC incidence rates (12.8 per 100,000 in women and 6.3 per 100,000 in men) and, between 1977 and 1984, had incidence rates of *S*. Typhi infection ranging from >90 to 121 cases per 100,000 (10). Vaccination of large cohorts of school children between 1980 and 1985 with live oral typhoid vaccine lowered the annual rates in Santiago by 1990 (10). Luria-Bertani (LB) agar Lennox (Difco Laboratories, Detroit, MI, USA) was prepared according to the package instructions and used to grow *S*. Typhi strains overnight at 37°C (18, 19, 28).

### Stem cell isolation, culture media, and the establishment of the HODGM model

**Figure 1:**
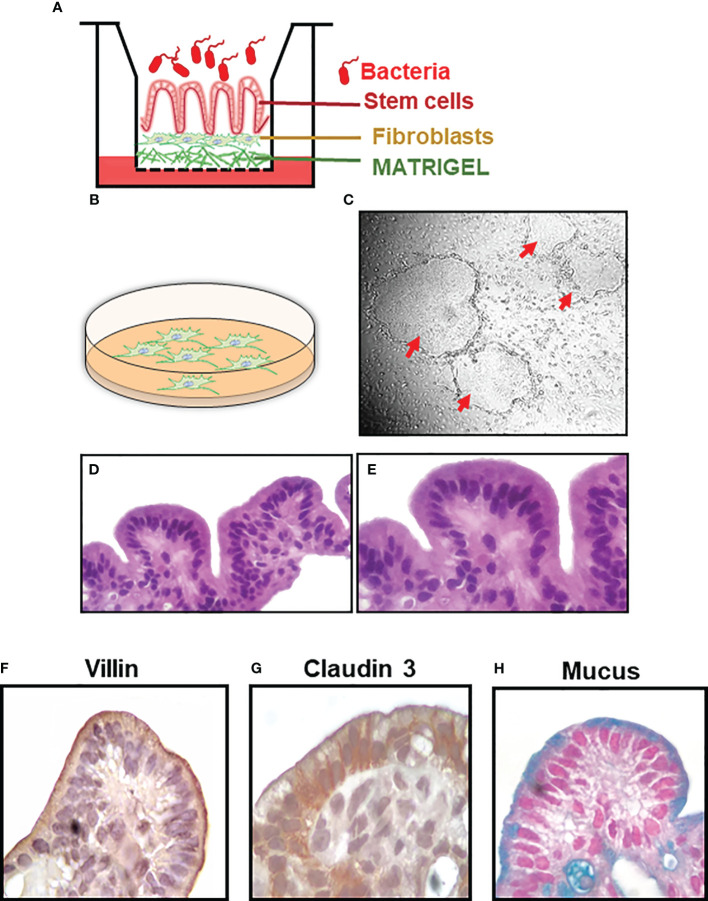
Establishment of the HODGM model. **(A)** Cartoon of the model. **(B)** Feeder layer of fibroblasts. **(C)** Co-culture of stem cells and feeder cells in a laminin-rich matrix (Matrigel) and the formation of 2-D colonies (red arrows). Cells from a differentiated model, H&E, at **(D)** lower and **(E)** higher magnification. Immunochemical staining of the HODGM model to detect the presence of **(F)** microvilli using an anti-villin mAb, **(G)** tight junctions using an anti-claudin 3 mAb, and **(H)** mucus using Alcian Blue at higher magnification.

No animal models faithfully recapitulate *S*. Typhi infection (20, 21). To partially address this shortcoming, we used a HODGM model developed by our group (**Figure 1A**). Gallbladder stem cell (SC) isolation, culture, and establishment of the HODGM model were performed according to previous protocols with few modifications (29–31). Briefly, gallbladder tissues were rinsed with chelation buffer (2% sorbitol, 1% D-sucrose, 1% bovine serum albumin fraction V (BSA), 10 μg/ml Gentamicin and 250 ng/ml Amphotericin in Dulbecco’s Phosphate buffered saline) (Sigma-Aldrich-Aldrich, St Louis, MO, USA)), minced, and incubated with PBS/2 mM EDTA for 30 minutes to release SC clusters. The SC clusters were then co-cultured with 3T3-J2 γ-irradiated mouse embryonic fibroblasts in 6-well plates coated with Matrigel and containing intestinal Organoid Culture Medium (DMEM/F-12 medium supplemented with 10% L-WRN conditioned medium (32), 100 U/mL penicillin, 100 µg/mL streptomycin, 50 µg/mL gentamicin, 2 mM L-glutamine, 1 mM sodium pyruvate, 100 μM MEM-Non-Essential Amino-Acids, 10 mM HEPES buffer, 5 μg/ml insulin (Sigma-Aldrich-Aldrich, St Louis, MO, USA), 0.4 μg/ml 3,3’,5-triiodo-L-thyronine (T3, Sigma-Aldrich), 1.8 10^-4^ M adenine (Sigma-Aldrich), 5 μg/ml transferrin (Sigma-Aldrich), 10^-10^ M cholera toxin (Sigma-Aldrich), 500 nM A-83-01 (Sigma-Aldrich), 10 µM Y-27632 Rock inhibitor (Biogems, Westlake Village, CA), 50 ng/mL epidermal growth factor (EGF) recombinant human protein (ThermoFisher, Waltham, MA, USA), 0.4 μg Hydrocortisone (Sigma-Aldrich), 1 µM Jagged-1 peptide (AnaSpec, Fremont, CA, USA), and 10 mM Nicotinamide (Sigma-Aldrich)) (**Figure 1B**). After 10-15 days, SC colonies (**Figure 1C**) were dissociated with Trypsin-EDTA (0.25%) (Invitrogen), and 500,000 SC were plated onto 0.4μm transwells coated with Matrigel and containing 200,000 γ-irradiated fibroblasts (**Figure 1A**). Monolayers were cultured to confluence and subsequently differentiated for 3-4 days in differentiation media (intestinal Organoid Culture Medium without L-WRN conditioned medium and Nicotinamide) under an air-liquid interface (ALI) (**Figure 1A**).

### *S.* Typhi infection

The HODGM models were incubated for 5 hours at 37°C in DMEM media (without antibiotics) in the presence of one of the 13 *S*. Typhi strains isolated from acutely (n=6) and chronically (n=7) infected patients at a multiplicity of infection (MOI) of 50 (19, 28, 33). HODGM models with media only were used as controls. After 5 hours, supernatants were collected for the detection of cytokines. Cells were also collected from the transwell membrane to prepare whole-cell lysates and/or to extract mRNA. Alternatively, cell-containing transwells were fixed and used for immunochemical staining.

### Preparation of the tissues from the HODGM model for histology and immunohistochemistry

The following primary anti-human antibodies were used for immunohistochemistry: (1) rabbit anti-claudin 3 polyclonal antibody (1:100, (ThermoFisher) and (2) anti-villin monoclonal antibody (mAb) (clone CWWB1, 1:30, Vector, Burlingame, CA, USA). Alcian Blue (pH 2.5) Stain Kit (Vector) was used to detect mucin. Immunohistochemistry was performed as previously described with a few modifications (28, 33, 34). Briefly, 1 mL of 10% paraformaldehyde (PFA) was added above and underneath the transwell for 2 to 48 hours at room temperature. After fixation, using forceps, the membrane was gently cut from the transwell, added into biopsy cassettes, and transferred to the Histology Facility to be embedded in paraffin blocks, and cut into 5 μm sections. The sections were then consecutively rinsed with Histo-Clear (National Diagnostics, Atlanta, GA, USA), 100% ethanol, 95% ethanol, 75% ethanol, and finally rehydrated in PBS for 10 min. For histological staining, tissue sections were stained with hematoxylin and eosin (H&E) and examined under a light microscope for morphological changes. For immunochemical staining, histological sections were autoclaved at 120 °C for 30 seconds (Pascal chamber, DAKO) in sodium-citrate buffer (Invitrogen). After washing, the sections were treated with PBS, 3% H_2_O_2_, blocked with PBS/0.5% Tween-20/5% horse serum, and incubated for 2 hours with primary antibodies at room temperature. Specific antigens were detected by incubating the sections with anti-Mouse/Rabbit horseradish peroxidase-labeled antibody (ImmPRESS Universal Antibody Kit, Vector) for 30 minutes at room temperature. Immunostaining was visualized using DAB peroxidase-chromogen reaction (ImmPACT DAB kit, Vector).

### Cytokine production following *S*. Typhi infection

Supernatants from the upper and lower chamber were harvested 5 hours after adding the *S*. Typhi strains to the cultures and kept at -20°C until assayed. Levels of interleukin (IL)-8 in the culture supernatants were measured by commercial ELISA (eBioscience, San Diego, CA). Levels of IL-6, IL-18, monocyte chemoattractant protein-1 (MCP-1), thymic stromal lymphopoietin (TSLP), and tumor necrosis factor (TNF)-α were quantified by Meso Scale Discovery (MSD, Gaithersburg, MD) multiplex-assay. ELISA and MSD measurements were carried out following the manufacturer’s instructions.

### Western blot

Western blots were performed according to previous protocols with few modifications (33). Briefly, after 5 hours of incubation with the *S*. Typhi strains, the transwells were gently rinsed with PBS, and their membranes cut. The membranes were then added into power ceramic beads tubes (Qiagen, Valencia, CA) containing 1 mL of ready-to-use RIPA buffer (Sigma-Aldrich) supplemented with a cocktail of protease and phosphatase inhibitors (Sigma-Aldrich) (33) to prepare the whole-cell lysates. The mixture was briefly vortexed and incubated on ice for 30 min. After incubation, the bead-beating tubes were placed in a Bullet Blender Homogenizer (Next Advance, Troy, NY, USA) for 30 seconds to lyse residual cells. The lysates were then clarified by centrifugation at 10,000 x *g* for 15 min at 4 °C, transferred to new microfuge tubes, and the pellets discarded. The cell lysates’ protein concentration was determined using a Thermo Scientific Pierce Micro BCA Protein Assay kit (Thermo Scientific, Rockford, IL, USA). The following primary anti-human antibodies were used for Western Blot: (1) rabbit anti-phospho S6 (Ser240/244) mAb (1:2000) (clone D68F8), (2) rabbit anti-pan S6 mAb (1:1000) (clone 5G10), (3) rabbit anti-phospho MAPK(p-p44/42 extracellular signal-regulated kinase (ERK) 1/2) mAb (1:1000) (clone D13.14.4E), (4) rabbit anti-pan MAPK(p-p44/42 Erk1/2) polyclonal antibody (1:1000), (5) rabbit anti-phospho YAP (Ser109) mAb (1:600) (clone E5I9G), (6) rabbit anti-pan YAP mAb (1:1000) (clone D8H1X), (7) rabbit anti-phospho Axl (Tyr702) mAb (1:1000) (clone D12B2), (8) rabbit anti-pan Axl mAb (1:1000) (clone C44G1), (9) rabbit anti-phospho Akt (Ser473) mAb (1:2000) (clone D9E), (10) rabbit anti-pan Akt mAb (1:1000) (clone C67E7), (11) rabbit anti-phospho mTOR (Ser2448) polyclonal antibody (1:1000), (12) rabbit anti-pan mTOR polyclonal antibody (1:1000), (13) rabbit anti- Enhancer of zeste homolog 2 (EZH2) mAb (1:1000) (clone D2C9), (Cell Signaling, Danvers, MA, USA), (14) mouse anti-interferon regulatory factor 1 (IRF1) mAb (1:500) (clone 686703), and (15) rabbit anti-glyceraldehyde-3-phosphate dehydrogenase (GAPDH) polyclonal antibody (1:15000) (R&D Systems, Minneapolis, MN). Blots were incubated with their specific pan- or GAPDH-specific antibodies to normalize the cell lysate protein loading. Band intensity was quantitated using Image J software (https://imagej.net/software/imagej).

### Assessment of MAPK-regulated transcription factors

A highly sensitive non-radioactive ELISA-based assay, the transcription factor assay (TransAM, Active Motif, Carlsbad, CA, USA), was used to monitor the activation of cellular Myc (c-Myc), myocyte enhancer factor-2 (MEF2), and activating transcription factor-2 (ATF-2) transcription factors. Procedures were performed according to the manufacturer’s instructions. Briefly, 4-6 μg of cell lysate was used to detect cellular c-Myc, MEF2, and ATF-2 phosphorylation. The lysate containing the transcription factor was incubated with an oligo mixture immobilized in the well, followed by incubation with primary and secondary HRP-conjugated antibodies used to quantify the amount of activated transcription factors by spectrophotometry.

### RNA isolation and NanoString gene expression profiling

RNA was isolated from transwell membranes according to previous protocols with few modifications (19, 33, 35). Briefly, membranes were cut, added into power ceramic beads tubes (Qiagen, Valencia, CA) containing 1 mL RNA RLT Buffer (RNeasy Micro kit, Qiagen, Valencia, CA, USA), and beaten in a Bullet Blender Homogenizer for 30 seconds; total cellular RNA was then isolated according to the manufacturer’s instructions. RNA concentrations were measured on a NanoDrop 1000 spectrophotometer (Thermo Fisher) and 75ng of RNA per sample were used for assaying on the NanoString nCounter platform, using the Human Immunology V2 Panel to profile 579 genes covering the core pathways and processes of the immune response. Data were processed using the NanoString nSolver software v4.0 comparing acute vs. uninfected and chronic vs uninfected conditions, using within chip uninfected controls and recommended settings (including initial QC, negative control count thresholding using mean +/- 2 standard deviations, positive control normalization using default settings but excluding housekeeping genes with counts below 100 as well as G6PD and GAPDH that were found to vary in expression in our assays, and calculation of expression ratios of acute or chronic vs. uninfected). After normalization, expression values were compiled into a single matrix for differential expression (DE) analysis. DE genes were estimated using R packages Limma (36) & NanoTube (37), with chip-to-chip variation modeled into the analysis. DE genes meeting a p-value>0.01 were used to identify enriched Gene Ontologies for biological processes using the R package ClusterProfiler v4.0 (38).

### Statistical analysis

All statistical tests were performed using Prism software (version 9, GraphPad Software, La Jolla, CA). Comparisons between acutely and chronically patient-derived *S*. Typhi strains were carried out by two-tailed nested-t-test to account for the repeated measures within the groups. The nested t-test utilizes a mixed model approach and can accommodate missing values. In this test, data values are organized into subcolumns, with the selection of these subcolumns assumed to be random. However, the grouping factor (*e.g.*, acute vs. chronic strains) remains fixed. Therefore, the test examines the differences between these two groups while considering the random selection of strains within each group. Thus, this model effectively captures variations both within and between subcolumns, presenting them as variance and standard deviation (the square root of variance). (https://www.graphpad.com/guides/prism/latest/statistics/statinterpreting-results-nested-t-.htm). Mixed-effects models were used to compare multiple groups. Correlations used the two-sided Pearson Product Moment tests. Principal Component Analysis (PCA) was executed using ClustVis web tools (39) as described previously (40–42). Briefly, we applied unit variance scaling to rows, and we used singular value decomposition (SVD) with imputation to calculate principal components. *P* values < 0.05 were considered statistically significant when comparing the 2 groups of *S*. Typhi strains.

## Results

### Establishment of the HODGM model

*S.* Typhi is a human-restricted pathogen, and no animal models faithfully recapitulate *S*. Typhi infection (20, 21). Thus, to test our hypotheses that host molecular pathways activated by *S*. Typhi strains derived from acutely and chronically infected patients would differ, we established a HODGM model using gallbladder tissues from otherwise healthy individuals undergoing laparoscopic cholecystectomy. Clusters containing SC were isolated from the explants and co-cultured with fibroblasts in plates coated with Matrigel containing a cocktail of growth factors (**Figure 1B**). Once enough stem cells (SCs) were obtained for experimentation, colonies of undifferentiated SCs (**Figure 1C**) were dissociated and plated onto transwells to facilitate their conversion into fully differentiated epithelial cells. After differentiation, lineage, morphology, and functionality markers were used to characterize the HODGM model. As shown in **Figure 1**, the HODGM model displayed a well-differentiated cell phenotype as demonstrated by the presence of polarized columnar epithelial cells (**Figures 1D, E**) expressing lineage marker for absorptive gallbladder cells (villin) (**Figure 1F**). We also observed positive immunostaining for claudin-3, which demonstrated the formation of tight junctions (**Figure 1G**). Finally, we confirmed the functionality of our model via Alcian Blue mucus staining (**Figure 1H**), which additionally confirmed the presence of goblet cells along the epithelial layer.

### *S*. Typhi strains derived from acutely infected patients induced MAPK activation differently than *S*. Typhi strains derived from chronically infected patients

**Figure 2:**
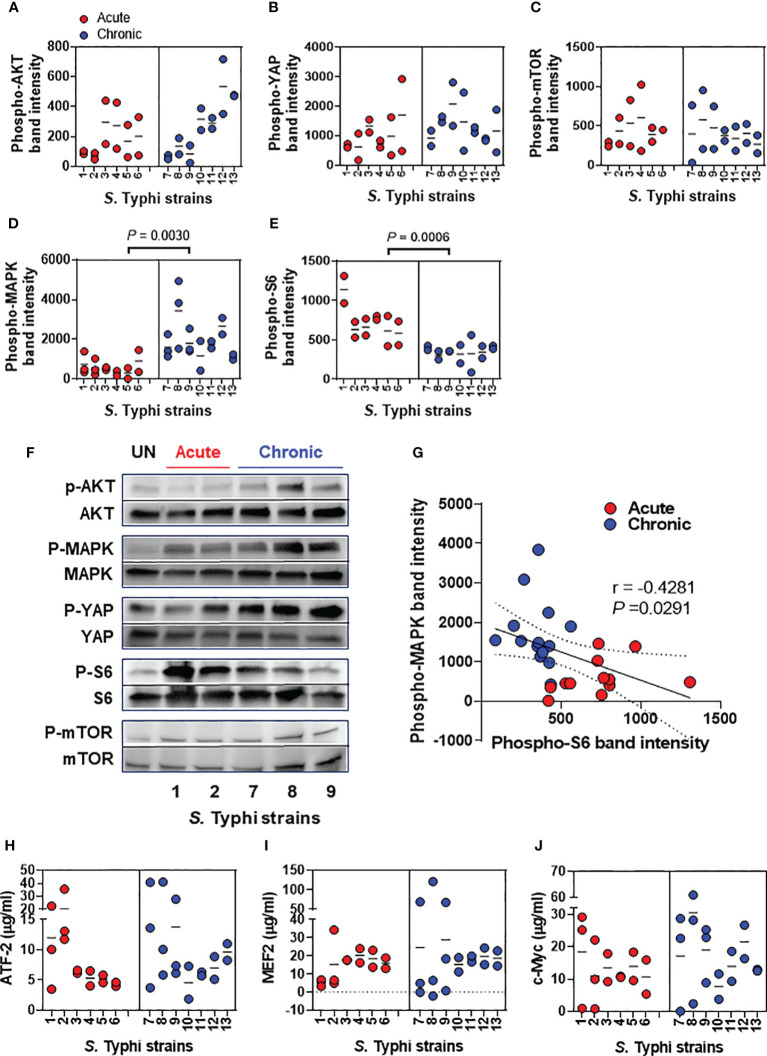
*Salmonella* modulation of gallbladder epithelial cell molecular pathways. Epithelial cells from HODGM model were exposed to 13 *S*. Typhi strains isolated from acutely infected (n=6, 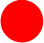) or chronically (n=7, 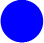) infected individuals. HODGM models cultured with media only were used as negative controls. After 5 hours, the cells were harvested, lysed, and phosphorylation of **(A)** AKT, **(B)** YAP, **(C)** mTOR, **(D)** MAPK, and **(E)** S6 were detected by western blot. Data are representative of the net responses observed in at least 4 experiments. Net responses were calculated by subtracting the responses of the controls (media) from those in cells exposed to *S*. Typhi. Each dot is the average of 2 technical independent replicates. The density was normalized to the corresponding pan kinase. Two-tailed nested-t-tests were used to account for the repeated measures within the groups. Dotted lines represent the mean responses. **(F)** Shown is a representative immunoblot analysis of protein lysates. Results from uninfected media controls (UN) and acute and chronic strains are displayed **(G)** Correlation between phosphorylation levels of S6 and MAPK using the combined data after exposure to *S.* Typhi strains isolated from acutely and chronically infected individuals. The solid line represents the trendline. Dashed lines represent 95% confidence intervals. Shown are the coefficient of determination “r” and the “*P*” value. Correlations used the two-sided Pearson Product Moment tests. **(H-J)** MAPK regulated transcription factors **(H)** Phospho-ATF-2, **(I)** Phospho-MEF2, and **(J)** Phospho-c-Myc were detected by ELISA-based assays, TransAM assays. Data are representative of the net responses observed in 5 experiments. Net responses were calculated by subtracting the responses of the controls (media) from those in cells exposed *S*. Typhi. Each dot is the average of 2 technical independent replicates. Two-tailed nested-t-test was used to account for the repeated measures within the groups. *P* values < 0.05 were considered statistically significant. Dotted lines represent the mean responses.

A recent report has shown that *S*. Typhimurium-mediated activation of AKT and MAPK pathways drives the transformation of susceptible gallbladder cells (11); however, little information is available concerning the activation of these pathways during the early stages of the *S*. Typhi infection. In addition, there is no reports on the possible differential activation of AKT and MAPK pathways by *S*. Typhi strains derived from acutely and chronically infected patients. Thus, we started our studies by evaluating several host transcription factors involved in cell proliferation and differentiation, including AKT, YAP, mTOR, MAPK, and S6 (**Figures 2A-F**). We found that, albeit at different levels, the exposure of the HODGM model to any of the *S*. Typhi strains resulted in increased phosphorylation of AKT, YAP, mTOR, MAPK, and S6 transcription factors as compared with negative controls (media only) (**Figure 2**). Interestingly, *S*. Typhi strains derived from acutely and chronically infected patients mediated the activation of S6 and MAPK differently (**Figures 2D, E**). While MAPK phosphorylation was low in cells exposed to *S*. Typhi strains derived from acutely infected patients, it was significantly higher in cells exposed to *S*. Typhi strains derived from chronically infected patients (**Figures 2D, F**). In contrast, while S6 phosphorylation was high in cells exposed to *S*. Typhi strains derived from acutely infected patients, it was significantly lower in cells exposed to *S*. Typhi strains derived from chronically infected patients (**Figures 2E, F**). As expected, we observed a significant inverse correlation between MAPK and S6 expression (**Figure 2G**). Of note, we found no statistically significant differences in the levels of AKT, YAP, and mTOR transcription factors when comparing HODGM models exposed to *S*. Typhi strains derived from acutely infected patients and those derived from chronically infected patients (**Figures 2A-C**). Since the MAPK antibodies used in our experiments detect endogenous levels of pan and phosphorylated p44/42 MAP kinase (Erk1/Erk2) proteins, we then evaluated three Erk1/Erk2 substrates, i.e., c-Myc, MEF2, and ATF-2 transcription factors. By using cell lysates prepared 5 hours after bacterial exposure, we observed that the levels of c-Myc, MEF2, and ATF-2 proteins were not statistically different when comparing cells exposure to *S*. Typhi strains derived from acutely and chronically infected patients (**Figures 2H-J**). In sum, although no differences were found in the levels of intranuclear c-Myc, MEF2, and ATF-2 transcription factors, these results suggest that *S*. Typhi strains derived from acutely and chronically infected patients induced MAPK and S6 signaling pathways differentially.

### HODGM model production of cytokines after exposure to *S*. Typhi strains from acutely or chronically infected patients

**Figure 3:**
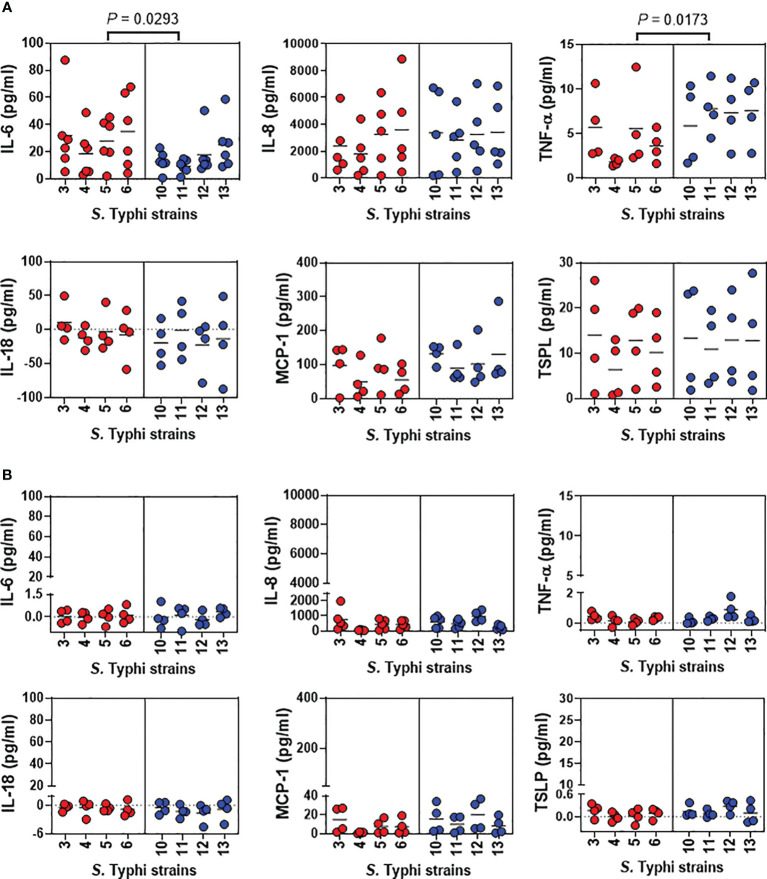
Detection of cytokines after exposure of the HODGM model to different *S*. Typhi strains. Epithelial cells from the HODGM model were exposed to 8 *S*. Typhi strains isolated from acutely infected (n=4, 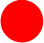) or chronically (n=4, 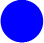) infected individuals. HODGM models cultured with media only were used as negative controls. After 5 hours, the supernatants were collected from the **(A)** upper and **(B)** lower chambers of the HODGM model to measure IL-6, IL-8, TNF-α, IL18, MCP-1, and TSLP cytokines. Data are representative of the net responses observed in 4 independent experiments. Net responses were calculated by subtracting the responses of the controls (media) from those in cells exposed *S*. Typhi. Each dot is the average of 2 independent replicates. Two-tailed nested-t-tests were used to account for the repeated measures within the groups. *P* values < 0.05 were considered statistically significant. Dotted lines represent the mean responses.

Previous studies from our group have shown that *S*. Typhi strains exhibiting high degrees of homology but with minor variations in gene expression can elicit dissimilar cytokine production by human gut epithelial cells (18, 19). Thus, we next investigated epithelial cell-producing cytokines after 5 hours of exposure to *S*. Typhi strains derived from acutely and chronically infected patients. We measured IL-6, IL-8, TNF-α, IL-18, MCP-1, and TSLP secretion in the supernatants collected from the top and bottom of the transwells of the HODGM model. For supernatants collected from the transwell top, we found that albeit at different levels, the exposure to any of the *Salmonella* strains, resulted in changes in cytokine secretion compared to negative controls (**Figure 3A**). Interestingly, we observed that *S.* Typhi strains derived from acutely and chronically infected patients triggered different patterns of TNF-α and IL-6 secretion. Exposure to *S.* Typhi strains derived from acutely infected patients induced a significantly higher secretion of IL-6 compared to strains derived from chronically infected patients (**Figure 3A**). In contrast, exposure to *S.* Typhi strains derived from chronically infected patients prompted a significantly higher secretion of TNF-α compared to *S.* Typhi strains derived from acutely infected patients (**Figure 3A**). Even though we found no correlations between the levels of TNF-α and IL-6, TNF-α levels directly and inversely correlated with the expression of phosphor-MAPK and phospho-S6, respectively (**Supplementary Figure 1**). Although not statistically significant, the exposure to *S.* Typhi strains derived from chronically infected patients triggered an increased secretion of MCP-1 compared to *S.* Typhi strains derived from acutely infected patients (**Figure 3A**). No significant differences in the secretion of IL-8, IL-18, or TSPL were observed among the strains (**Figure 3A**). Of note, in the supernatants collected from the bottom of the transwells and regardless of the *S*. Typhi origin, we found marginal or no increases in the cytokine levels as compared to controls (**Figure 3B**). Thus, we speculate that the differences in MAPK and S6 signaling pathways observed between *S*. Typhi strains derived from acutely and chronically infected patients might be related to differences in cytokine signaling pathways.

### *S.* Typhi strains derived from acutely and chronically infected patients induced distinct alterations in immunologically relevant host gene expression

**Figure 4:**
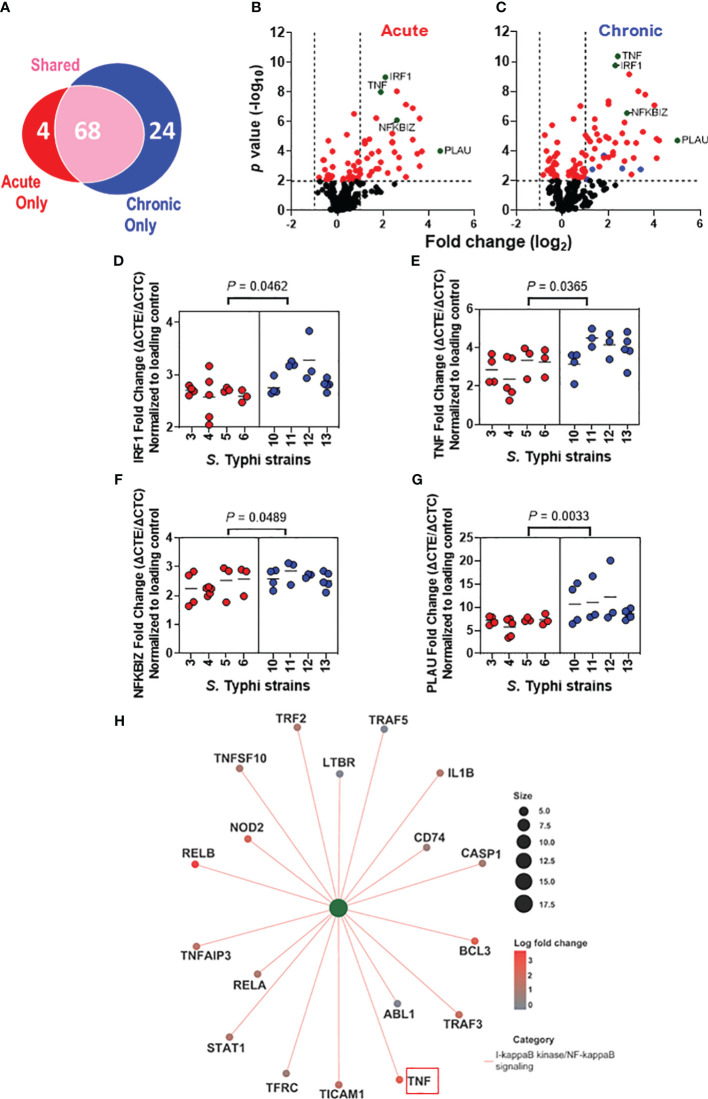
Distinct alterations in the immunologically related host gene expression after exposure to *S*. Typhi strains derived from acutely and chronically infected patients. Epithelial cells from HODGM model were exposed to 8 *S*. Typhi strains isolated from acutely infected (n=4, 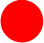) or chronically (n=4, 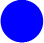) infected individuals, and gene expression was assessed by Nanostring assay using the nCounter Human Immunology V2 profiling Panel. **(A)** Venn diagram showing the number of genes significantly upregulated that are unique or shared among the two *S*. Typhi strain groups. **(B, C)** Volcano plots depicting differentially expressed gene P value (-log_10_) as a function of fold change(log_2_) in HODGM models exposed to S. Typhi strains isolated from acutely **(B)** or chronically **(C)** infected individuals. Red dots (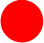) indicate *P* values of <0.01. Green dots (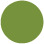) indicate genes that were significantly increased in cultures exposed to S. Typhi strains derived from chronically infected patients compared to those derived from acutely infected patients. Blue dots (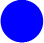) indicate genes with expressions >2 fold higher than the controls that were upregulated only by the *S*. Typhi strains derived from chronically infected individuals. **(D-G)** Individual gene expression data of **(D)** IRF1, **(E)** TNF, **(F)** NFKBIZ, and **(G)** PLAU. Data are representative of 3 individual experiments. Each dot symbolizes the log2 fold change of the samples exposed to *S*. Typhi strains and those cultures with media only (controls). The two-tailed nested-t-test was used to account for the repeated measures within the groups. **(H)** Network diagram with associated data to color nodes to visualize relationships. I-kappaB kinase/NF-kappaB signaling category.

We next assessed the effect of the *S*. Typhi origin on gene expression patterns in the HODGM model exposed to *S*. Typhi strains derived from acutely and chronically infected patients using the NanoString nCounter platform (**Figure 4**). Transcript levels of 579 predefined immunologically relevant genes were assessed using nCounter Human Immunology V2 Panel. Regardless of the strain assignment group, 96 differentially expressed genes were identified after exposure to *S*. Typhi (**Figure 4A**) compared to the controls (media only). After deconvolution of the data based on *S*. Typhi origin, we found that *S*. Typhi strains derived from acutely infected patients upregulated 72 genes after adjusting the *P*-value (adjusted *P* values < 0.01) (**Figures 4A, B**). On the other hand, *S*. Typhi strains derived from chronically infected patients upregulated 92 genes after adjusting the *P*-value (adjusted *P* < 0.01) (**Figures 4A, C**). Of note, we found a significantly higher proportion of unique genes upregulated by the chronic *S*. Typhi strains (*i.e*., 24) than those upregulated by acute *S*. Typhi strains (*i.e*., 4) (*P* < 0.0001, two-sided Chi-square, or *P* = 0.0002, two-sided Fisher’s Exact test). Out of the 24 genes upregulated only by the chronic *S*. Typhi strains, 5 genes (i.e., C-C Motif Chemokine Ligand 22 (CCL22), suppressor of cytokine signaling 1 (SOCS1), Signal Transducer And Activator Of Transcription 1 (STAT1), TNF Alpha Induced Protein 3 (TNFAIP3), and TNF Receptor Superfamily Member 9 (TNFRSF9)) were >2 fold higher than the controls (**Figure 4C**, 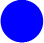). We also observed that while nearly all statistically significant genes upregulated by the acute *S.* Typhi strains were similarly upregulated by the chronic *S*. Typhi strains compared to the controls, higher magnitude changes were induced by the chronic *S*. Typhi strains compared to those induced by acute *S*. Typhi strains (**Figures 4A-C**). Among these statistically significantly upregulated genes that the expressions were >2 fold higher than the controls, four genes (*i.e.*, IRF1, TNF, NFKB Inhibitor Zeta (NFKBIZ), and Plasminogen Activator, Urokinase (PLAU)) were significantly increased in cultures exposed to *S*. Typhi strains derived from chronically infected patients compared to those derived from acutely infected patients (**Figures 4B, C**, 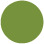). Increased IRF1 expression was further confirmed by western blot (**Supplementary Figures 1A, C**). Of note, TNF-α results are consistent with our MSD data showing an increased secretion of TNF-α after exposure to *S*. Typhi strains derived from chronically infected patients as opposed to those acutely infected (**Figure 3A**). No differentially downregulated genes were observed among cells exposed to *S*. Typhi strains and those cultured with media only (controls), after adjusting the *P*-value (adjusted *P <*0.01).

Considering the essential role of IRF1 in protecting against invading pathogens (43–45) and tumorigenesis (46–48), we examined the expression of EZH2, a substrate of IRF1 (45, 49). We found a significantly increased expression of IRF1 and EZH2 in cultures with *S*. Typhi strains derived from chronically infected patients compared to cultures with strains derived from acutely infected patients (**Supplementary Figure 2**). More importantly, we found a striking significant direct correlation between the expression of IRF1 and either the expression of Ezh2 or the levels of TNF-α (**Supplementary Figures 2E, F**).

Next, we performed pathway enrichment analysis to visualize data relationships in a network diagram (*i.e.*, cnetplot) (**Figure 4H**). In these analyses, we used differentially expressed genes with adjusted cutoffs *of P* < 0.01 to run gene ontology (GO) categories and get the pathway lists. We accounted for in the pathway analysis the 4 genes significantly increased in cultures exposed to *S*. Typhi strains derived from chronically infected patients compared to those derived from acutely infected patients (*e.g*., TNF, IRF1, NFKBIZ, and PLAU) (**Figures 4B, C**), and the genes exclusively upregulated by either *S*. Typhi strains derived from acutely (n=4) or chronically (n=24) infected patients (**Figure 4A**). Based on the counts of the genes, I-kappaB kinase/NF-kappaB signaling pathway was exclusively enriched in cultures with chronic *S*. Typhi strains when compared to the controls, as 8/18 genes (e.g., ABL1, CASP1, CD74, LTBR, STAT1, TFRC, TNFAIP3, and TRAF2) (*P* < 0.0001) enriched for this pathway were DE only in chronically infected patients (**Figure 4A**) and not enriched in cultures with acute *S*. Typhi strains (**Figure 4H**). The process includes a cascade of signals downstream within the cell through the I-kappaB-kinase (IKK)-dependent activation of NF-kappaB signaling. Of note, when compared to the controls, GO enrichment analysis revealed that there were 4 and 5 enriched pathways for acute and chronic *S*. Typhi strains, respectively, including 3 of 4 genes described in **Figures 4B**, C (*e.g*., TNF, IRF1, and NFKBIZ) contained in these GO categories (**Supplementary Figures 3, 4**).

These data further confirm that *S*. Typhi strains derived from acutely and chronically infected patients induce distinct early molecular alterations in host gallbladder cells, including in the abundance of genes whose expression is involved in immunological responses.

### Unsupervised principal component analysis and associations between the analytical variables

**Figure 5:**
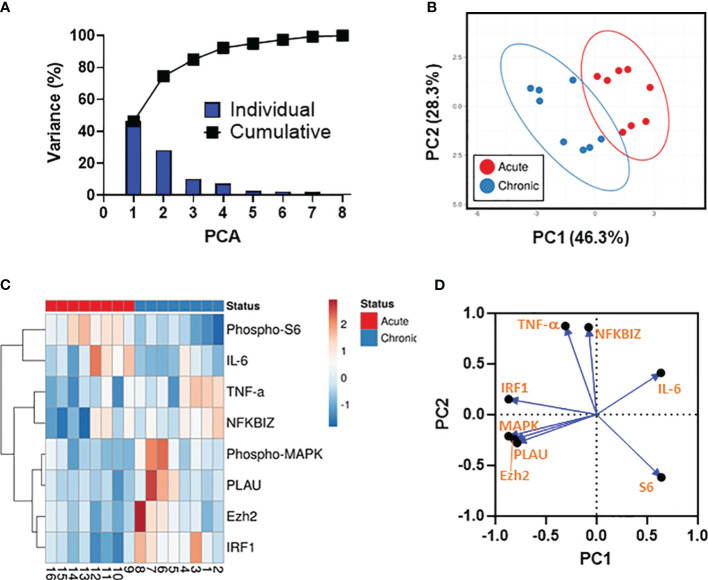
Data Integration using Principal Component Analysis (PCA). **(A)** The percent variation is plotted for each component (bars) and cumulatively (line). **(B)** PCA. Unit variance scaling is applied to rows; SVD with imputation is used to calculate principal components. X and Y axis show principal component 1 (PC1) and principal component 2 (PC2) that account for 46.3% and 28.3% of the total variance, respectively. Prediction ellipses are such that with a probability 0.95, a new observation from the same group will fall inside the ellipse. n= 16 data points. **(C)** Dendogram. Rows are centered; unit variance scaling is applied to rows. Rows are clustered using correlation distances and average linkages. Columns are clustered using binary distance and average linkage. 8 rows, 16 columns. **(D)** PCA loadings plot showing the distributions of the analytical variables.

We next used PCA to dimensionality reduce the data, enabling the investigation of associations between the analytical variables. To this end, all results involving analytes showing statistically significant differences between the 2 strain groups (*i.e*., phospho-MAPK, phosphor-S6, NFKBIZ, PLAU, Ezh2 (protein), IRF1 (protein), TNF-α, and IL-6) were merged for the combined analysis, generating a matrix of 8 analytes and 8 *S*. Typhi strains from two independent experiments. Only cases with complete data for all 8 analytical variables were included in the analyses. These analyses revealed that the first (PC1) (46.3%) and second (PC2) (28.3%) components accounted for most of the total (74.6%) variance and were able to clearly separate the 2 strain groups (**Figures 5A, B**). Based on the clustering tightness, we observed that analytical variables upregulated by strains derived from acutely infected patients displayed tighter clustering than their counterparts from chronically infected patients (**Figure 5B**). Next, we performed a hierarchical clustering analysis (**Figure 5C**). PCA arrangement of the analytical variables demonstrated three main clusters (i.e., (I) phospho-MAPK, PLAU, Ezh2, and IRF1, (II) TNF-α and NFKBIZ, and (III) phosphor-S6, and IL-6) indicating that the variables within the cluster are in somehow related (**Figure 5C**). Interestingly, we also observed that the clades for phospho-S6 and IL-6 had the highest height suggesting a substantial dissimilarity with the clades for NFKBIZ, phospho-MAPK, PLAU, TNF-α, Ezh2, and IRF1 (**Figure 5C**). To expand and confirm these analyses, we used a loading plot to gain further insights into the relationships between individual variables. As shown in **Figure 5D**, the vectors for IL-6 and PLAU form a nearly 180° angle, indicating that these variables are negatively correlated. Moreover, the vectors for IL-6 and phosphor-S6 are shaped at almost a right angle, suggesting that these variables are likely uncorrelated (**Figure 5D**). Collectively, these results demonstrated that multiple differential mechanisms might be involved in the host responses to the 2 *S*. Typhi strain groups, including negative feedback loops that can inhibit the activity of defined signaling pathways.

### Host responses after exposure of the HODGM to *S.* Typhi strains with epidemiological linkage isolated between 2017 and 2019

**Figure 6:**
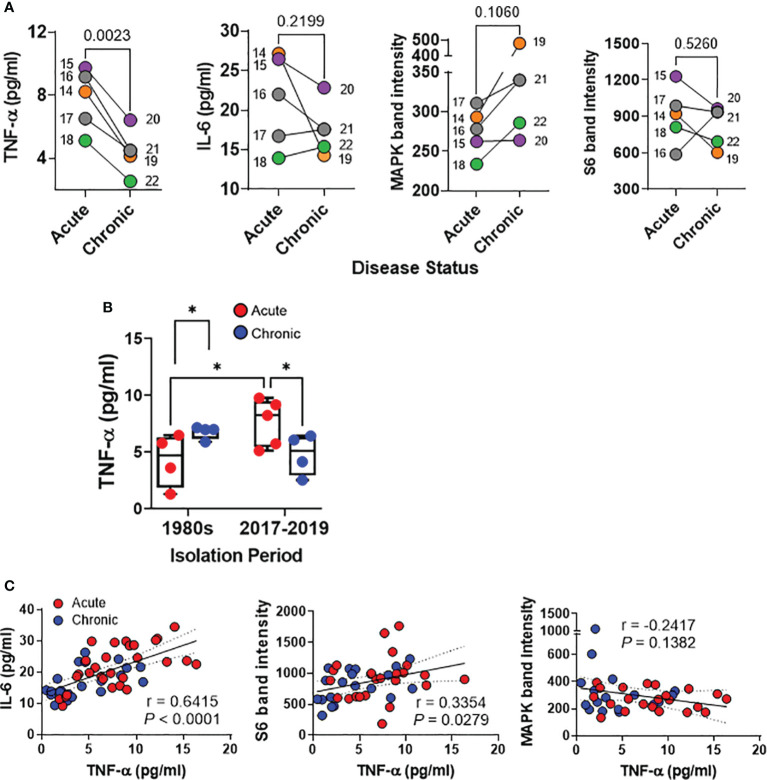
Detection of host responses after exposure of the HODGM model to *S*. Typhi strains with epidemiological linkage isolated between 2017and 2019. Epithelial cells from the HODGM model were exposed to 9 *S*. Typhi strains isolated from acutely infected (n=5) or chronically (n=4) infected individuals. HODGM models cultured with media only were used as negative controls. **(A)** After 5 hours, the supernatants were collected from the upper chambers of the HODGM model to measure IL-6 and TNF-α cytokines. Cells were also harvested, lysed, and phosphorylation of S6 and MAPK, were detected by western blot. Data are representative of the net responses observed in 2 independent experiments. Net responses were calculated by subtracting the responses of the controls (media) from those in cells exposed to *S*. Typhi. Each dot is the average of 2 experiments with 2-3 independent replicates. Colors denote the strain linkage: (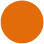) 14 & 19, (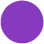) 15 & 20, (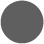) 16-17 & 21, (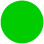) 18 & 22. Two-tailed paired t-tests were used to account for the strain linkage. **(B)** Production of TNF-a by the HODGM model exposed to 17 *S*. Typhi strains isolated in two distinct periods, 1980s from 4 acutely (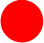), and 4 chronically (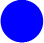) infected individuals and between 2017 and 2019 from 5 acutely (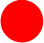), and 4 chronically (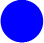) infected individuals. Mixed-effects model was used to compare multiple groups. The data are representative of 4 experiments with 2 independent replicates. **(C)** Correlation between TNF-a levels and IL-6, S6, and MAPK after exposure to *S.* Typhi strains isolated from acutely (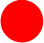) and chronically (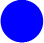) infected individuals. The data are representative of 2 experiments with 2-3 independent replicates The solid line represents the trendline. Dashed lines represent 95% confidence intervals. Shown are the coefficient of determination “r” and the “*P*” value. Correlations were determined using the two-sided Pearson Product Moment tests. *P* values < 0.05 were considered statistically significant.

Recent studies utilizing whole genome sequencing (WGS) and phylogenetic analysis have revealed a striking similarity among *S*. Typhi strains isolated from acute cases of typhoid fever in Santiago, Chile, between 2017 and 2019 -a period marked by low incidence of typhoid fever- and isolates from acute cases collected during the 1980s when typhoid fever was hyperendemic in Chile (24). Additionally, WGS investigations have yielded nearly identical genomes among isolates derived from chronic carriers and their associated acute typhoid cases from the years 2017 to 2019 (26). Hence, it is reasonable to hypothesize that the variations observed in host immune responses in the HODGM model herein are not attributable to genotypic and phylogenetic differences among the strains. Instead, they are more likely the result of disparities in bacterial gene expression within the gallbladder environment, underscoring the fundamental role of strain origin. To gain deeper insights into the impact of the origin of *S*. Typhi isolates (acute typhoid fever versus chronic gallbladder carriage) on host responses, we assessed nine well-characterized isolates collected in Santiago between 2017 and 2019, 4 isolates from chronic carriers and 5 from their corresponding typhoid cases (**Supplementary Table 1**) (26). Furthermore, to minimize potential biases associated with preconceived knowledge of the origin of the isolate before conducting the experiments, the disease status (acute or chronic) was not disclosed by the investigators involved in the specimen collection, who had access to clinical data, until the experiments were performed, the database locked, and the results analyzed. After 5-hour exposure to the isolates, we collected cells and supernatants from the top of the transwells and analyzed four analytes, i.e., IL-6 and TNF-α production, as well as MAPK and S6 expression, which had previously exhibited differences between *S*. Typhi isolates from acute cases and chronic carriers in the 1980s’ collection (**Figures 2**, **3**). We found that *S*. Typhi strains derived from acutely infected patients elicited significantly higher TNF-α secretion than those derived from chronically infected patients (**Figures 6A, B**). While not statistically significant, exposure to *S*. Typhi strains from acute cases tended to also induce greater IL-6 secretion compared to strains from chronic carriers (**Figures 6A, B**). Conversely, exposure to *S*. Typhi strains from chronically infected patients exhibited a trend towards higher expression of phospho-MAPK compared to strains from acutely infected patients (**Figure 6A**). No significant differences were observed in the expression of phospho-S6 among the strain groups (**Figure 6A**). Surprisingly, we discovered a significant direct correlation between the levels of TNF-α and either IL-6 (r = 0.6415, *P <*0.0001) or S6 (r = 0.3354, *P* = 0.0279) (**Figure 6C**). No correlation was found between the expression of phospho-MAPK and the levels of TNF-α (**Figure 6C**). It is important to note that levels of TNF-α in cultures exposed to isolates from acutely infected patients collected between 2017 and 2019 were significantly higher than in cultures exposed to isolates from acutely infected patients collected in the 1980s (**Figure 6B**). Interestingly, we found no significant differences between the TNF-α levels among the chronic strains isolated in 1980s and those isolated during 2017-2019 (**Figure 6B**) suggesting that contrary to acute strains, the TNF-α levels tend to reach a plateau during chronic infection. As mentioned above, minimal, or negligible increases in cytokine levels, in comparison to controls, were detected in the supernatants collected from the bottom of the transwells, irrespective of the origin of *S.* Typhi (**Supplementary Figure 5**). Taken together, these findings provide additional evidence to reinforce our hypothesis regarding the significant influence of the origin of the *S*. Typhi isolates on host immune responses. Furthermore, these results indicate a potential correlation between the levels of TNF-α and the time that the clinical isolate was subjected to selective pressure within the human gallbladder.

## Discussion

Understanding the early events triggering the transition of acute to chronic *S*. Typhi infections may help to identify new tools to combat typhoid carriage and long-term *S*. Typhi reservoirs in typhoid-endemic areas. Heretofore, little has been known about the early molecular mechanisms involved in the interactions between gallbladder epithelial cells and *S*. Typhi.

We have found that *S*. Typhi strains derived from acutely and chronically infected patients mediated the activation of S6 and ERK-MAPK differently. We recorded that MAPK phosphorylation was significantly lower in HODGM models exposed to *S*. Typhi strains derived from acutely infected patients than in HODGM models exposed to *S*. Typhi strains derived from persons with chronic gallbladder infection. Conversely, S6 phosphorylation was significantly higher in HODGM models exposed to *S*. Typhi strains derived from acutely infected patients than after exposure to *S*. Typhi strains derived from chronically infected patients. The ERK pathway, also known as the p44/p42 MAPK pathway, is an important signaling pathway regulating cell proliferation, differentiation, and survival (50). Its hyperactivation has been shown to play a significant role in cancer development and progression (50). Notably, the MAPK signaling pathway is also activated in GBC tissues from Chilean (51) and Indian (52) patients, underscoring the importance that it might have in driving gallbladder transformation. Remarkably, S6 represents an essential downstream effector of the ERK-MAPK signaling pathway (53). S6 is a ribosomal protein that regulates protein synthesis activated by the mTOR signaling pathway (54, 55). It has been reported that p44/p42 MAPK pathway can inhibit the phosphorylation of tuberous sclerosis complex 2 (TSC2) protein, a regulator of mTOR (56). Of note, the mTOR pathway can inhibit the p44/p42 MAPK pathway. This inhibition can occur through the phosphorylation and inactivation of the mTORC1 complex (57), a protein complex that controls the initiation of S6 protein translation (58). Also, p70S6 kinase has been shown to inhibit the p44/p42 MAPK pathway by directly phosphorylating and inhibiting the activity of the MAPK kinases MEK1 and MEK2 (59), enzymes that activate the p44/p42 MAPK pathway (60). p70S6 kinase is an enzyme that catalyzes the phosphorylation of the S6 protein and is activated by the mTOR signaling pathway (61, 62). Thus, as expected, the relationship between S6 and p44/p42 MAPK transcription factors is complex and may depend on the specific context and multiple mechanisms, including mTORC1-MAPK feedback loops (63, 64).

Another important finding in this manuscript is the observation of direct and inverse associations between the secretion of TNF-α and the expression of phospho-MAPK and phospho-S6 proteins, respectively. These results align with prior research, underscoring the importance of p44/p42 MAPK pathways in the modulation of TNF-α production (65). Moreover, Takai and colleagues have elegantly showcased that TNF-α triggers IL-6 synthesis via the p44/p42 MAPK pathway (66). However, a discernible discrepancy emerges within our HODGM models utilizing isolates from the 1980s. While IL-6 secretion demonstrated increased levels in HODGM exposed to *S*. Typhi strains from acute illness cases compared to those from chronic gallbladder carriers, intriguingly, no apparent correlations emerged between IL-6 and either TNF-α, phospho-MAPK, or phospho-S6 proteins. Notably, high expression of IL-6 has been associated with tumor differentiation, local invasion, and poorer survival among patients with GBC (67, 68). An explanation for these variations could be the lack of epidemiological linkage between the strains from acute cases and those from chronic carriers obtained in the 1980s. To test this hypothesis, we used strains isolated in Chile between 2017 and 2019, with known epidemiological strains linkage among acute cases and chronic carriers (26). These latter studies, performed in a blinded fashion, validated the direct correlation between IL-6 levels and TNF-α production within our HODGM models exposed to *S*. Typhi strains. These findings corroborate the functional interdependence of TNF-α and IL-6. It is also important to emphasize that besides its role in inflammation, IL-6 is required for tissue healing through its induction of intestinal epithelial proliferation (69). The PCA arrangements underscored the close interplay between TNF-α and NFKBIZ, a nuclear inhibitor of the NF-κB pathway that governs the TNF pathway (70). Thus, changes in TNF-α production may be required to establish and maintain long-term infection. Of note is the demonstration with *in vivo* mouse models infected with non-flagellated mutant *Salmonella* strains, indicating an association between the expression of the phase 1 filament subunit protein FliC, a flagella protein, and the induction of TNF-α production. Remarkably, re-establishing FliC expression in these non-flagellated mutant strains effectively upregulates TNF-α (71). It is worth mentioning that FliC plays a crucial role in the pathogenesis of enteric infections caused by flagellated bacteria such as *Salmonella*. It represents an essential virulent factor as it is directly involved in the motility, adherence, invasion, establishment, and dispersal of pathogens during infection. However, once inside the cell, flagella are no longer required for the motility of the bacteria and are downregulated to evade host immune responses, including activation of pro-inflammatory cytokines (72, 73). Thus, an unexplored question in this manuscript is the potential contribution of differing pathogen-associated molecular patterns (PAMPs) in strains, such as FliC, in the initial activation of distinct signaling pathways. It is conceivable that variations in the composition or abundance of PAMPs among the *S*. Typhi strains could lead to differential activation of innate receptors such as Toll-like receptors (TLRs) (*e.g*., lipoproteins, TLR2; lipopolysaccharide, TLR4; and flagellin, TLR5) (74), thereby influencing downstream signaling events. It is important to note that TLR5 contributes to p38 MAPK but not to the p44/p42 MAPK pathway upon flagellated bacteria challenge (75), suggesting another mediator is involved in the activation (*e.g.*, cytokines). Future studies focusing on elucidating these mediators could provide valuable insights and significantly enhance our understanding of the observed differences and correlations in the signaling pathways.

Of note, we demonstrated that the expression of IRF1 transcription factor increases in cells exposed to *S.* Typhi strains derived from chronically infected patients as opposed to the strains from acutely infected patients. Studies with ascidians, who share a common ancestor with vertebrates, suggested that IRF represents one of the most conserved components of innate immunity (76). Indeed, IRF1 signaling is essential for generating mucosal Th17 cell responses to *S*. Typhimurium in mice (44). Remarkably, we found a statistically significant correlation between IRF1 and Ezh2, and tighter PCA clustering of IRF1, phospho-MAPK, PLAU, and Ezh2. EZH2 belongs to the polycomb group genes (PcGs) family, a group of critical epigenetic regulators that repress transcription (77). Inhibition of EZH2 can decrease the expression of inflammatory genes via IRF1 (78). In contrast, its overexpression has been linked with various malignancies, including prostate cancer, ovarian cancer, and breast cancer (79). Similar to Ezh2, PLAU is also implicated in developing multiple cancer types (80, 81). Moreover, we observe a statistically significant correlation between IRF1 and TNF-α. Previous studies have demonstrated that IRF1 is critical for chronic TNF responses (82, 83). Notably, our study revealed no significant differences in TNF-α levels between chronic strains isolated in the 1980s and those isolated during 2017-2019, in contrast to the results observed with acute strains. This suggests that IRF1 might modulate TNF-α levels based on disease status (chronic vs. acute). Indeed, IRF1 exhibits a dual role, e.g., combating invading pathogens (43–45) and contributing to tumorigenesis (46–48). These findings imply that *S.* Typhi strains obtained from chronically infected patients trigger distinct molecular pathways compared to those from acutely infected patients.

Here, we have also validated our hypothesis on the critical role of the origin of *S*. Typhi isolates on host responses. When comparing Chilean isolates obtained from acute cases during two different periods, 2017-2019, and the 1980s, we observed that levels of TNF-α in cultures exposed to isolates collected between 2017 and 2019 were significantly higher than in cultures exposed to isolates collected in the 1980s. Recent studies using WGS and phylogenetic analysis have uncovered a remarkable similarity between *S*. Typhi strains obtained from acute cases of typhoid fever in Santiago, Chile, between 2010 and 2016 - a period of low typhoid fever incidence - and strains collected during the 1980s when typhoid fever was hyperendemic in Chile (24). This discovery strongly suggests that the isolates in acute cases in 2017-2019 primarily originated from chronic typhoid carriers, in contrast to the *S*. Typhi strain isolates from the 1980s, which were likely to arise from other acutely infected patients. Thus, we hypothesize that in our HODGM models, isolates from acute cases during the 1980s may be less adapted to establish the infection compared to strains isolated between 2017 and 2019. As proposed by others, the increased adaptability of the isolates from 2017 to 2019 may enable further evasion of host immunity while concurrently inducing hyperinflammation continuously, akin to what is observed in chronic infection with opportunistic pathogens such as *Pseudomonas aeruginosa* (84). Contrary to other infections with intracellular microorganisms, during acute disease, TNF-α production favors the spread of *S*. Typhimurium in mice (73). Concurrently and/or alternatively, these differences might be linked to the evolution and spread of *S*. Typhi during the two distinct periods: 2017 to 2019 and the 1980s. It is tempting to hypothesize that in the 1980s, when susceptible hosts were abundant, various strains of *S*. Typhi coexisted. However, in the years 2017 to 2019, following systematic typhoid vaccination and improved sanitary conditions, hosts became more resistant to the infection. As a result, the same variants would engage in intense competition, wherein the variant demonstrating superior person-to-person transmission capability would likely emerge dominant (85). Our findings also further reinforce our prior reports, indicating that genetically engineered live attenuated *S*. Typhi vaccine candidates elicit elevated TNF-α levels compared to wild-type *S*. Typhi strains (18). Moreover, our results are in agreement with studies by Devaraj and colleagues showing that while sequence analysis of multiple isolates from acute cases and chronic carriers across various geographical locations did not reveal distinct clustering based on the chronic carrier or acute infection status of patients, chronic isolates exhibited distinct phenotypes from acute infection isolates (86). Chronic carriage isolates were observed to form thicker biofilms than acute isolates (86), indicating a further adaptation for biofilm formation on gallstone surfaces, which promotes persistence in the human gallbladder (87). Other research from Baker’s group similarly highlighted that *S*. Typhi carriage wasn’t limited to any specific genotype but driven by selective pressure within the human gallbladder, underscoring the significance of the isolate’s origin (88).

Finally, the authors are aware of the HODGM model limitations. Our model does not fully recapitulate the human *in vivo* microenvironment. This is a consequence of the model representing only epithelial cells in two dimensions. Human gallbladder environments are more complex, containing immune cells, bile, and on many occasions, gallstones. Our model also only represents early local immune responses. Systemic and chronic infections cannot be represented in our model. We also acknowledge some variation in how the HODGM models recognize and respond to different strains within the same group. Based on our previous studies demonstrating that closely related *S*. Typhi strains can elicit somewhat different innate cell responses in the human intestinal mucosa (18, 19), we anticipated such variations. Hence, we deliberately selected multiple strains within each group for testing. Of note, the statistical analyses were conducted using a two-tailed nested t-test. This conservative approach accommodates repeated measures within the groups, thereby mitigating the impact of any single strain on the overall results. For instance, it is possible to forego subgrouping and simply employ all observations in a standard t-test. However, this method treats all observations within each group as entirely independent, disregarding the variability between strains. Incorporating all observations in a t-test, essentially compares the disparity in group means to the level of variation within each group, assuming multiple independent strain measurements. This abundance of measurements might erroneously inflate the perceived accuracy of the strain mean for each group, potentially magnifying even minor differences between acute and chronic strains as “significant.”

In conclusion, the results presented here expand our understanding of the largely unknown molecular mechanisms involved in the early interactions of gallbladder epithelial cells with *S*. Typhi and evidence important differences in the host responses based on whether the isolates are derived from acute typhoid fever patients or chronic carriers. Our data also argue for additional feedback mechanistic studies on the MAPK pathway and signaling pathways involved in cytokine production following *S*. Typhi infection.

## Data availability statement

The original contributions presented in the study are publicly available. The GEO accession number for the Nanostring assays is GSE253700 (https://www.ncbi.nlm.nih.gov/geo/query/acc.cgi?).

## Ethics statement

The studies involving humans were approved by University of Maryland Institutional Review Board. The studies were conducted in accordance with the local legislation and institutional requirements. The ethics committee/institutional review board waived the requirement of written informed consent for participation from the participants or the participants’ legal guardians/next of kin because a study exemption has been approved (#HP-00077485).

## Author contributions

RS-G: Conceptualization, Data curation, Formal analysis, Investigation, Methodology, Project administration, Supervision, Validation, Writing – original draft. HC: Data curation, Formal analysis, Methodology, Writing – review & editing. AB: Data curation, Methodology, Resources, Writing – review & editing. MI: Data curation, Formal analysis, Methodology, Writing – review & editing. JH: Data curation, Methodology, Resources, Writing – review & editing. RL: Data curation, Methodology, Resources, Writing – review & editing. HT: Data curation, Formal analysis, Methodology, Writing – review & editing. AD: Data curation, Formal analysis, Methodology, Writing – review & editing. JB: Data curation, Methodology, Resources, Writing – review & editing. AF: Data curation, Funding acquisition, Methodology, Writing – review & editing. ML: Conceptualization, Data curation, Formal analysis, Funding acquisition, Methodology, Writing – review & editing. MS: Conceptualization, Data curation, Formal analysis, Funding acquisition, Methodology, Writing – original draft.
