# Supplementary material for: Early host immune responses in a human organoid-derived gallbladder monolayer to Salmonella Typhi strains from patients with acute and chronic infections: a comparative analysis
Source: Front Immunol. 2024 Mar 12;15:1334762. doi: 10.3389/fimmu.2024.1334762 (PMC10963533; doi:10.3389/fimmu.2024.1334762)
Supplement: Supplementary file 1 [file Presentation_1.pdf]

Early Host Immune Responses in a Human Organoid-Derived Gallbladder Monolayer to Salmonella Typhi Strains from Patients with Acute and Chronic Infections: A Comparative Analysis

Rosângela Salerno-Goncalves, Haiyan Chen, Andrea C. Bafford, Mariana Izquierdo, Juan Carlos Hormazábal, Rosanna Lagos, Hervé Tettelin, Adonis D'Mello, Jayaum S. Booth, Alessio Fasano, Myron M. Levine, and Marcelo B. Sztein

Supplementals

Supplemental Table 1. Description of *S*. Typhi serovar strains used in the manuscript

Disease Status	ID	Strain	Genotype	Origin	Linkage	Source	References
Acute	1	Ty2	4.1*	Russia, 1948/WRAIR**		-	(22)
	2	ISP1820	-	Chile, 1988		Blood Culture	(25)
	3	2251/83	3.1	Chile, 1983		Blood Culture	(24)
	4	2262/83	2.3.4	Chile, 1983		Blood Culture	(24)
	5	1994/83	2	Chile, 1983		Blood Culture	(24)
	6	1366/83	2	Chile, 1983		Blood Culture	(24)
	14	314-2017_CI	3.5	Chile, 2017	314-2017_CO_03	Blood Culture	(26)
	15	1521-2017_CI	1.2.1	Chile, 2017	1521-2017_CO_04	Blood Culture	(26)
	16	1698-2017_CI	2	Chile, 2017	1698-2017_CO_06	Blood Culture	(26)
	17	424-2019_CI	2	Chile, 2019	1698-2017_CO_06	Blood Culture	(26)
	18	2027-2019_CI	3.5	Chile, 2019	2027-2019_CO_04	Blood Culture	(26)
Chronic	7	Quailes	3.0.1	Maryland, USA, 1958		Gallbladder	(27)
	8	2704/81	-	Chile, 1981		Stools	This study
	9	2709/81	-	Chile, 1981		Stools	This study
	10	4060/82	-	Chile, 1982		Stools	This study
	11	3555/82	-	Chile, 1982		Stools	This study
	12	3129/82	-	Chile, 1982		Stools	This study
	13	4048/82	-	Chile, 1982		Stools	This study
	19	314-2017_CO_03	3.5	Chile, 2019		Stools	(26)
	20	1521-2017_CO_04	1.2.1	Chile, 2019		Stools	(26)
	21	1698-2017_CO_06	2	Chile, 2019		Stools	(26)
	22	2027-2019_CO_04	3.5	Chile, 2019		Stools	(26)

*Wong et al. Nature Communications, 2016; ** WRAIR, Walter Reed Army Institute Hospital, Bethesda, Washington DC. Parent strain of the oral typhoid vaccine strain Ty21a

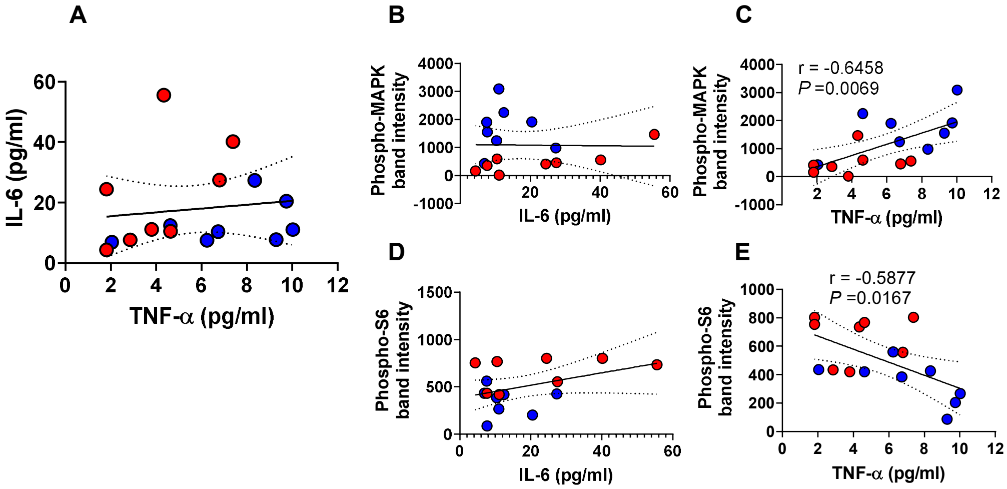

Supplemental Fig. 1. Correlation among S6 and MAPK signaling pathway markers and IL-6 and TNF-α cytokines after exposure to *S*. Typhi strains. Epithelial cells from HODGM model were exposed to 13 *S*. Typhi strains isolated from acutely (6, ●) or chronically (7, ●) infected individuals. Data are representative of the net responses observed in at least 2 experiments. Net responses were calculated by subtracting the responses of the controls (media) from those in cells exposed *S*. Typhi. Each dot is the average of 2 independent replicates. Trendlines (solid lines), the coefficient of determination "r" and "P" values are shown. Dashed lines represent 95% confidence intervals. Correlations used the two-sided Pearson Product Moment tests. *P* values < 0.05 were considered statistically significant

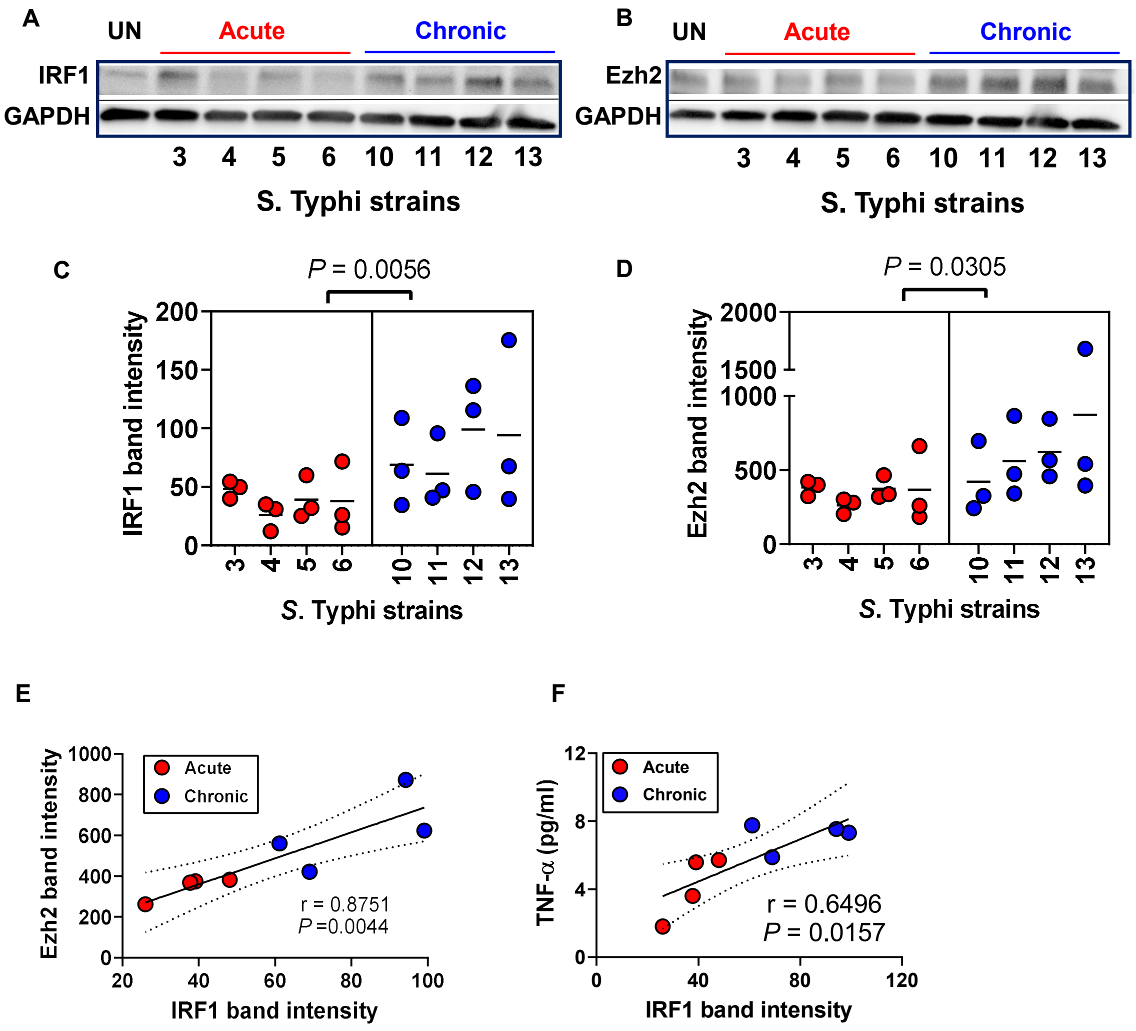

Supplemental Fig. 2. Association between IRF1 and Ezh2 gene expression. Epithelial cells from HODGM model were exposed to 8 *S*. Typhi strains isolated from acutely (4, ●) or chronically (4, ●) infected individuals. HODGM models cultured with media only were used as negative controls. After 5 hours, the cells were harvested, lysed, and the expression of (**A**) IRF1 and (**B**) Ezh2 were detected by western blot. Data are representative of the net responses observed in 3 independent experiments. Net responses were calculated by subtracting the responses of the controls from those in cells exposed *S*. Typhi. The density was normalized to the counterpart GAPDH specific antibodies. (**C-D**) Two-tailed nested-*t*-tests were used to account for the repeated measures within the groups. Correlations between the expression of IRF1 and either the expression of Ezh2 (**E**) or the levels of TNF-α (**F**) were performed on pooled data of 3 biological replicates from 3 independent experiments. Trendlines (solid lines), the coefficient of determination "r" and "*P*" values are shown. Dashed lines represent 95% confidence intervals. Correlations used the two-sided Pearson Product Moment tests. *P* values < 0.05 were considered statistically significant.

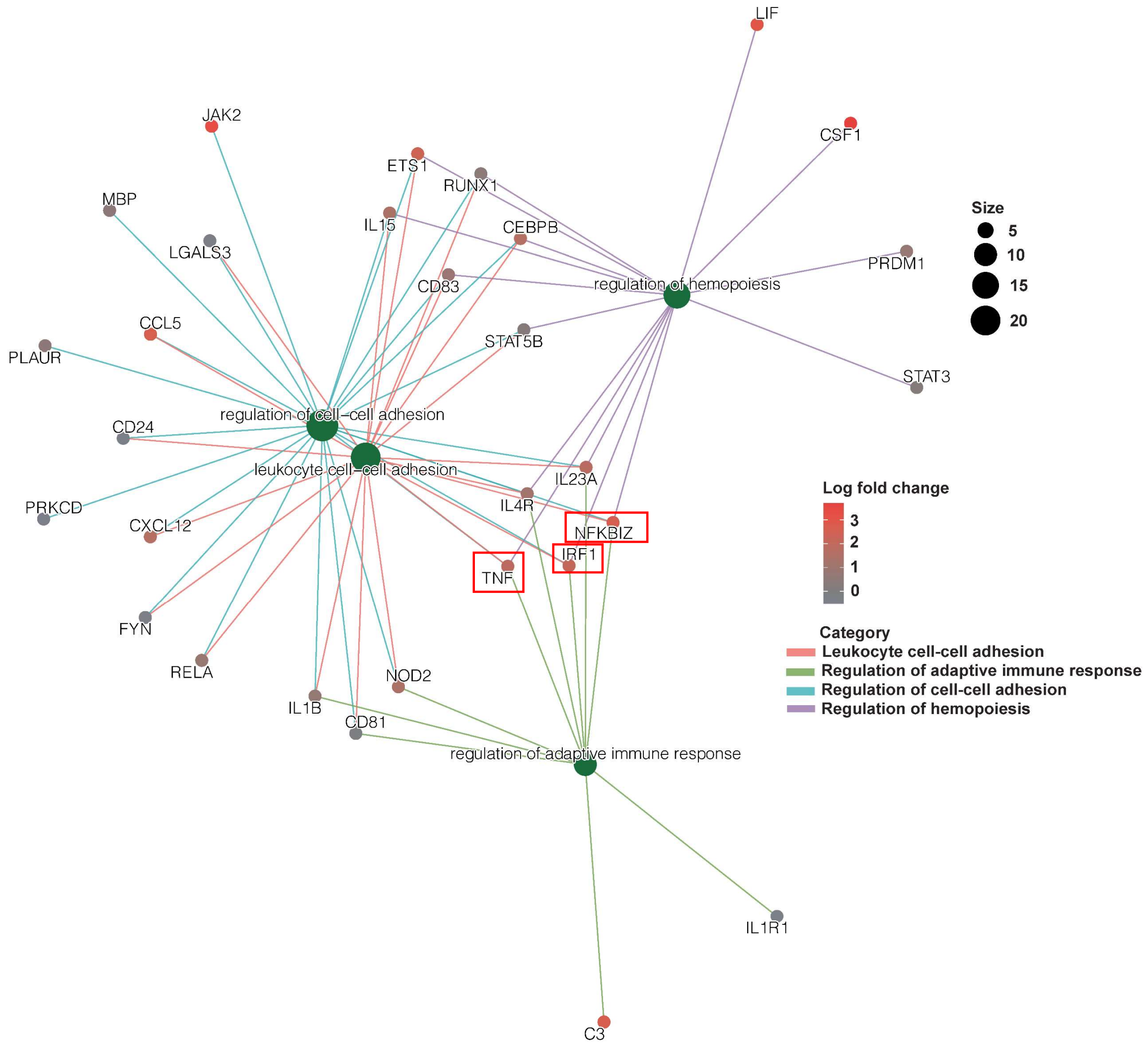

Supplemental Fig. 3. Network diagram with associated data to color nodes to visualize relationships. Gene ontology pathways enriched in cultures to exposed to *S*. Typhi strains derived from acutely infected patients compared to controls.

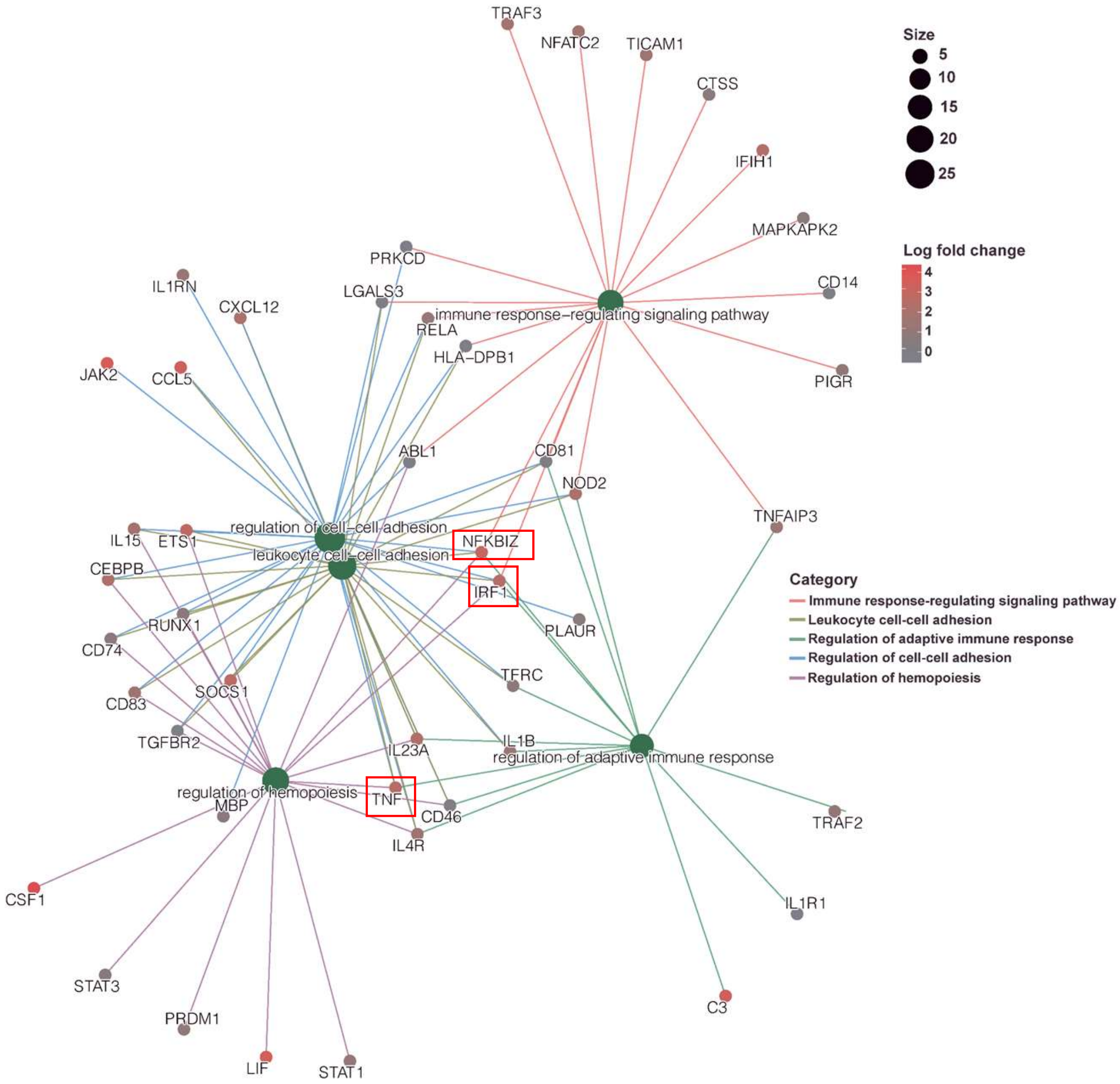

Supplemental Fig. 4. Network diagram with associated data to color nodes to visualize relationships. Gene ontology pathways enriched in cultures to exposed to *S*. Typhi strains derived from chronically infected patients compared to controls.

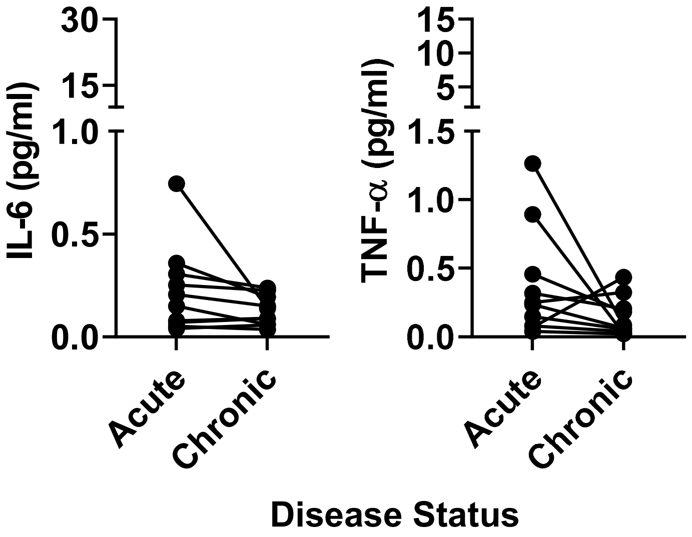

Supplemental Fig. 5. **Cytokine production at lower chambers of the HODGM after stimulation with different *S*. Typhi isolates collected between 2017 and 2019**. Epithelial cells from the HODGM model were exposed to 9 *S*. Typhi strains isolated from acutely infected (n=5) or chronically (n=4) infected individuals. HODGM models cultured with media only were used as negative controls. (A) After 5 hours, the supernatants were collected from the lower chambers of the HODGM model to measure IL-6 and TNF-α cytokines. Data are representative of the net responses observed in 2 independent experiments. Net responses were calculated by subtracting the responses of the controls (media) from those in cells exposed to *S*. Typhi. Each dot is the average of 2 independent replicates. Two-tailed paired t-tests were used to account for the strain linkage.
